# Data on undergraduate students’ self-regulation in online and blended learning environments during the COVID-19 pandemic in Indonesia

**DOI:** 10.1016/j.dib.2024.110066

**Published:** 2024-02-02

**Authors:** Irwanto Irwanto

**Affiliations:** Department of Chemistry Education, Universitas Negeri Jakarta, Jakarta 13220, Indonesia

**Keywords:** Self-regulation, Undergraduate students, Online learning, Blended learning, COVID-19 pandemic, Indonesia

## Abstract

The COVID-19 outbreak led higher education institutions to shift to distance learning. This article describes data on the self-regulation of undergraduate students in online and blended learning environments during the pandemic situation. A total of 577 students including 144 males and 433 females in Jakarta were recruited using a combination of purposive and snowball techniques. In this non-experimental voluntary survey design, the questionnaire designed by Barnard et al. [1] was adapted to collect data. The online survey was utilized to gather data on personal demographic information (15 items), goal setting (5 items), time management (3 items), environment structuring (4 items), help-seeking (4 items), task strategies (4 items), and self-evaluation (4 items). It was carried out during May and June 2023. The data were analyzed using frequency distributions and correlation analysis. The raw data is available in Excel format. The collected data offer new insights regarding students’ self-regulated online learning in terms of gender, academic year, age, daily internet usage, and more. The dataset will guide education policymakers on the role of self-regulatory skills for the success of technology-enhanced learning and educators to design educational programs to increase students’ academic performance in online and blended learning contexts.

Specifications Table

**Table:** 

Subject	Social Sciences – Education
Specific subject area	Higher Education Self-Regulation
Data format	Raw Analyzed
Type of data	Tables
How the data were acquired	Data were acquired through a self-administered survey questionnaire (Google Forms) and converted into .xlsx format for further analysis in SPSS. Using a mixture of purposive and snowball techniques, a total of 577 usable responses were acquired and analyzed quantitatively. The Online Self-Regulated Learning Questionnaire (OSLQ) designed by Barnard et al. [1] was adapted to reveal students’ self-regulation in both online and hybrid learning environments. The questionnaire comprised 24 close-ended questions with a five-degree Likert-type scale. Respondents should be 18 years of age or older to participate in the study. The participation of students was on a voluntary basis; thus, they could withdraw at any point without consequence. All personal data of respondents have been removed.
Description of data collection	Data were collected at the end of the second term of the 2022/2023 academic calendar and gathered using an online questionnaire between May and June 2023. The target respondents were university students aged between 19–25 years (20.26±1.41) in Jakarta, Indonesia, with a mobile device and internet access. They utilized online teaching platforms throughout this semester. The collection of this dataset took approximately one month to complete. The data are presented in raw and analyzed forms.
Data source location	Institution: Universitas Negeri Jakarta (UNJ) City/Town/Region: Jakarta Country: Indonesia Latitude and longitude: 6°10′30.00″ South, 106°49′35.76″ East
Data accessibility	Repository name: Mendeley Data Data identification number: 10.17632/7bsvck73ng.1 Direct URL to data: https://data.mendeley.com/datasets/7bsvck73ng/1

## 1 Value of the Data

• The survey dataset is useful because it contributes to mapping and identifying Indonesian students’ self-regulatory learning skills in tertiary education. This dataset is also useful for parents to find out how their children learn online and blended learning and can be used by parents to optimize informal education.
• The dataset is important because it can be used to investigate the impact of socio-demographic variables—i.e. faculty, gender, academic year, age, learning time before and during COVID-19, number of siblings, location of residence, source of tuition fees, cumulative average (GPA), daily internet usage, the purpose of internet usage, devices often use, parental education, and parental occupation—on students’ self-regulated online learning in the COVID-19 period and beyond.
• The dataset will be valuable for educators to understand students’ self-regulated learning behaviors and compare students’ self-regulatory competencies during and post-COVID-19 conditions.
• The collected survey dataset can be adapted for use in order to evaluate students’ self-regulation strategies in secondary education as well as improve pedagogical practices in secondary and higher education.
• The present dataset can be used by researchers, practitioners, policymakers, and education managers to enhance the quality of online and blended learning in order to promote students’ achievement results.
• The dataset can be used as a reference for other researchers to compare it with other datasets collected from other countries/territories or similar research exploring self-regulatory strategies in other disciplines.

## 2 Data Description

This dataset [2] is intended to offer valuable information on evaluating self-regulation throughout the COVID-19 emergency. The data will help readers understand the profile of students’ self-regulated learning (SRL) and explore the influence of the respondents’ demographic attributes on self-regulation. This dataset is also essential for comparison purposes; hence, future scholars worldwide can compare it with other datasets from other emerging nations. In addition, the data will help educators design effective interventions and innovative teaching methodologies to support technology-enhanced learning. The dataset provides useful information regarding students’ perceptions of SRL in online and blended learning contexts. The sample consisted of 577 active students who took courses in a hybrid class format. During remote learning, respondents used online platforms provided by UNJ or other Learning Management Systems (LMS), such as Moodle, Google Classroom, Edmodo, and other platforms chosen by their lecturers. Thus, they were familiar with synchronous and asynchronous online learning environments based on their blended learning experiences in the current and previous semesters.

The socio-demographic description of the respondents and their mean SRL scores grouped based on different characteristics are given in Table 1. Of the 577 students who participated in this study, nearly a third (*n*=183; 31.70%) came from the Faculty of Mathematics and Natural Sciences, followed by the Faculty of Languages and Arts (*n*=94; 16.30%), and the Faculty of Educational Psychology (*n*=83; 14.40%), while the lowest concentration came from Faculty of Engineering, with 5.40% of the sample (31 students). Among them, 75% (*n*=433) identified themselves as female while 25% (*n*=144) identified as male. This is a reflection of the gender distribution of undergraduate students at UNJ [3]. The university year of study distribution included 283 (49.00%) first-year, 163 (28.20%) second-year, 89 (15.40%) third-year, and 42 (7.30%) fourth-year students. Concerning age distribution, 430 (74.50%) students were between 18–20 years old, 142 (24.60%) were between 21–23 years old, and 5 (0.90%) were over 23 years old. The age range in the sample was 19–25 years, with a mean of 20.26 years (SD=1.41).

**Table 1:** Socio-demographic information of study respondents and mean SRL scores.

Category	Characteristic	Code	Frequency		SRL	
			F	%	M	SD
Faculty	Faculty of Economics	1	58	10.10	3.83	0.34
	Faculty of Education	2	55	9.50	4.05	0.36
	Faculty of Educational Psychology	3	83	14.40	3.70	0.44
	Faculty of Engineering	4	31	5.40	3.61	0.53
	Faculty of Languages and Arts	5	94	16.30	3.75	0.47
	Faculty of Mathematics and Natural Sciences	6	183	31.70	3.79	0.41
	Faculty of Social Sciences	7	36	6.20	3.80	0.45
	Faculty of Sport Sciences	8	37	6.40	3.88	0.40
Gender	Female	1	433	75.00	3.81	0.41
	Male	2	144	25.00	3.76	0.49
Academic year	First-year	1	283	49.00	3.84	0.45
	Second-year	2	163	28.20	3.76	0.42
	Third-year	3	89	15.40	3.75	0.43
	Fourth-year	4	42	7.30	3.78	0.42
Age	18–20 years old	1	430	74.50	3.79	0.44
	21–23 years old	2	142	24.60	3.83	0.41
	More than 23 years old	3	5	0.90	3.36	0.35
Learning time before COVID-19	Under 4h	1	126	21.80	3.72	0.46
	From 4 to 7h	2	299	51.80	3.78	0.41
	Over 7h	3	152	26.30	3.90	0.43
Learning time during COVID-19	Under 4h	1	228	39.50	3.67	0.42
	From 4 to 7h	2	288	49.90	3.87	0.43
	Over 7h	3	61	10.60	3.91	0.38
No. of siblings	One	1	230	39.90	3.78	0.43
	Two	2	203	35.20	3.77	0.42
	Three	3	98	17.00	3.86	0.44
	Four or more	4	46	8.00	3.82	0.49
Location of residence	Rural	1	61	10.60	3.96	0.39
	Suburban	2	182	31.50	3.76	0.42
	Urban	3	334	57.90	3.79	0.44
Source of tuition fees	Parents	1	366	63.40	3.78	0.45
	Scholarship	2	197	34.10	3.83	0.40
	Self-sponsored	3	14	2.40	3.80	0.37
Cumulative average (GPA)	3.5+	1	410	71.10	3.82	0.42
	3.0–3.49	2	134	23.20	3.75	0.45
	2.5–2.99	3	26	4.50	3.72	0.51
	Below 2.5	4	7	1.21	3.63	0.60
Daily Internet usage	Less than 1 hour	1	4	0.70	4.29	0.26
	1–6 hours	2	186	32.20	3.85	0.43
	7–12 hours	3	387	67.10	3.76	0.43
Purpose of Internet usage	Academic	1	11	1.90	4.36	0.32
	Non-academic	2	3	0.50	3.96	0.04
	All the above	3	563	97.60	3.78	0.43
Devices often use	Desktop PC	1	38	6.60	3.85	0.48
	Laptop	2	250	43.30	3.82	0.42
	Smartphone	3	283	49.00	3.77	0.44
	Tablet or iPad	4	6	1.00	3.80	0.36
Parental education	Primary school	1	31	5.40	3.89	0.29
	Secondary school	2	315	54.60	3.79	0.43
	Diploma	3	40	6.90	3.81	0.37
	Undergraduate	4	160	27.70	3.80	0.47
	Postgraduate	5	31	5.40	3.76	0.47
Parental occupation	Unemployed	1	74	12.80	3.80	0.42
	Self-employed	2	128	22.20	3.75	0.41
	Worker	3	99	17.20	3.83	0.41
	Private	4	198	34.30	3.79	0.44
	Government	5	78	13.50	3.86	0.49

In terms of hours of learning before the COVID-19 outbreak, about half (*n*=299) of students spent 4 to 7 hours, 152 spent more than 7 hours, and 126 spent less than 4 hours per day. Regarding the learning time allocated in the age of COVID-19, approximately half (*n*=288) of students also spent 4 to 7 hours, the rest spent more than 7 hours (*n*=152) and less than 4 hours per day (*n*=126). The majority of students had one (39.90%), two (35.20%), three (17.00%), and four or more (8.00%) siblings. With respect to residence location, 57.90% of respondents lived in urban, 31.50% in suburban, and 10.60% in rural areas. A large part of the survey respondents (*n*=366) reported that their parents sourced their tuition fees, while 197 students received scholarships and 14 students were self-sponsored. As for their cumulative average (GPA), most of the sample (*n*=410) were in the GPA group of 3.5 and above, accounting for 71.10% of the sample, followed by the 3.0–3.49 GPA group, with 23.20%, and only 7 had GPA below 2.5.

Out of these active students, two-thirds of respondents (67.10%) spent between seven and twelve hours a day online, 32.20% spent between one and six hours online, and only 0.70% spent less than one hour on the Internet. Besides, the purpose for using the Internet was categorized as 1.90% for academic purposes, 0.50% for non-academic purposes, while 97.60% reported using the Internet for all the options provided. With regard to devices often used, a total of 283 (49.00%) respondents were doing an online study through smartphones, 250 (43.30%) were using laptops, 38 (6.60%) were using desktop PCs, and only 6 (1.00%) were using tablets or iPads. As such, smartphones are the main tool for getting information during remote learning. Of those who self-selected to participate, 54.60% of students reported that their parents had graduated from secondary school at their highest education level, followed by undergraduate (27.70%), diploma (6.90%), elementary school (5.40%), and postgraduate (5.40%). The parental occupation sector informed that most of the respondents worked in the private sector with a percentage of 34.30%, followed by the self-employed at 22.20%, and workers at 17.20%. Other sectors (government) contributed 13.50%, while the unemployed was 12.80%.

When Table 1 examined, students from the Faculty of Education (M=4.05; SD=0.36), females (M=3.81; SD=0.41), those who were in their first year of study (M=3.84; SD=0.45), those aged between 21–23 years (M=3.83; SD=0.41), those who studied more than 7 hours a day before (M=3.90; SD=0.43) and during the pandemic (M=3.91; SD=0.38), those who had 3 siblings (M=3.86; SD=0.44), and those who lived in rural areas (M=3.96; SD=0.39) were the group most likely to self-regulate their learning. In addition, students who received scholarships (M=3.83; SD=0.40), those who had a GPA of 3.5 and above (M=3.82, SD=0.42), those who surfed the Internet less than 1 hour a day (M=4.29; SD=0.26), those who spent time online for academic purposes (M=4.36; SD=0.32), those who used a PC (M=3.85; SD=0.48), those whose parents had primary education (M=3.89, SD=0.29), and those whose parents worked in the government sector (M=3.86; SD=0.49) were the group that showed higher self-regulation than the others.

In the present study, the dataset consists of six OSLQ subscales as described in Table 2. The questionnaire was designed to collect students’ responses about the extent to which they were able to set standards and goals during online courses (Goal Setting; Items GS1–GS5), choose a convenient location for learning (Environment Structuring; ES1–ES4), adopt appropriate strategies, take notes, and do more problems in their online courses (Task Strategies; TS1–TS4), schedule and allocate time for learning every day (Time Management; TM1–TM3), seek help from peers, lecturers, or others to solve problems (Help-Seeking; HS1–HS4), and understand what they had learned (Self-Evaluation; SE1–SE4). The type of data collected is nominal, ordinal, and scale. The collected dataset is publicly available at https://data.mendeley.com/datasets/7bsvck73ng/1.

**Table 2:** The Cronbach's α coefficients.

Subscale	Code	No. of Items	Cronbach's α
Goal Setting	GS	5	0.78
Environment Structuring	ES	4	0.76
Task Strategies	TS	4	0.80
Time Management	TM	3	0.90
Help-Seeking	HS	4	0.72
Self-Evaluation	SE	4	0.80
All Subscales		24	0.94

After translating the original English version of OSLQ into Indonesian, a pilot test with 105 undergraduate students was conducted. The coefficients of Cronbach's alpha (α) obtained in the current study are presented in Table 2. The reliability of the OSLQ was calculated using Cronbach's α coefficient. Cronbach's α for the OSLQ was found to be 0.94, which indicated that the internal consistency of the instrument was considered acceptable. As can be seen in Table 2, Cronbach's α for all subscales ranged from 0.72 to 0.90, which implies high reliability [4].

After the data were collected, the researcher coded each statement and option by number. For example, the first statement on the Goal Setting subscale was coded GS1, then the option was coded as follows: strongly disagree was coded 1, as the minimum score, and strongly agree was coded 5, as the maximum score. A similar method was also followed for other statements on the related subscales. Each statement is assigned a code number as shown in Table 3. Data collected were then analyzed with IBM SPSS Statistics 25. Aiming to analyze and summarize quantitative data, such as mean, standard deviation, frequency, percentage, variance, skewness, and kurtosis, descriptive statistics were calculated. Descriptive statistics for all OSLQ items are displayed in Tables 3–4. Table 3 depicts the mean scores and standard deviations of each item. On Item GS1 regarding setting standards for assignments in online learning [1], students rated their self-regulation varying from 1 to 5 with a mean score of 4.10 and SD of 0.68. As observed in Table 3, the highest mean score was on Item ES1 “*I choose the location where I study to avoid too much distraction*” (M=4.38, SD=0.73) and the lowest was on Item TS4 “*I work extra problems in my online courses in addition to the assigned ones to master the course content*” (M=3.02, SD=0.92). This indicated that students are more able to self-regulate in terms of choosing a convenient location and avoiding distractions during online learning. In contrast, students rarely did extra tasks other than the tasks assigned in online classes. Overall, it is observed that the mean score of all items is above the midpoint of the questionnaire scale. Given the mean score obtained from the entire scale, which is 3.80 (SD=0.43), this represents that Indonesian students were medium to high online self-regulated learners. On a five-degree Likert scale, this measure is between neutral and agree. It indicated that students tend to have a slightly positive attitude towards self-regulated learning strategies. As observed in Table 3, the skewness and kurtosis values of each item are in the range of ±2 and ±7, respectively; thus, the data were normally distributed [5,6].

**Table 3:** Descriptive statistics on self-regulated learning.

Item	Min	Max	M	SD	Variance	Skewness		Kurtosis	
						Statistic	Std. Error	Statistic	Std. Error
GS1	1	5	4.10	0.68	0.46	−0.80	0.10	1.82	0.20
GS2	1	5	3.97	0.78	0.61	−0.79	0.10	0.83	0.20
GS3	1	5	3.68	0.92	0.84	−0.40	0.10	−0.50	0.20
GS4	1	5	4.14	0.73	0.53	−0.90	0.10	1.58	0.20
GS5	1	5	3.79	0.98	0.95	−0.91	0.10	0.49	0.20
ES1	1	5	4.38	0.73	0.54	−1.45	0.10	2.99	0.20
ES2	1	5	4.17	0.78	0.61	−0.90	0.10	0.85	0.20
ES3	2	5	4.20	0.71	0.51	−0.74	0.10	0.69	0.20
ES4	1	5	3.98	0.91	0.82	−0.93	0.10	0.67	0.20
TS1	1	5	3.88	0.91	0.84	−0.63	0.10	−0.01	0.20
TS2	1	5	3.11	1.08	1.17	0.08	0.10	−1.03	0.20
TS3	1	5	3.22	0.91	0.82	−0.08	0.10	−0.31	0.20
TS4	1	5	3.02	0.92	0.84	0.15	0.10	−0.35	0.20
TM1	1	5	3.56	0.92	0.84	−0.33	0.10	−0.45	0.20
TM2	1	5	3.55	0.94	0.88	−0.34	0.10	−0.55	0.20
TM3	1	5	3.79	0.81	0.66	−0.70	0.10	0.60	0.20
HS1	1	5	3.28	1.21	1.45	−0.26	0.10	−0.94	0.20
HS2	1	5	4.04	0.84	0.71	−1.03	0.10	1.27	0.20
HS3	1	5	3.88	0.95	0.90	−0.93	0.10	0.60	0.20
HS4	1	5	3.40	0.97	0.93	−0.26	0.10	−0.43	0.20
SE1	1	5	3.90	0.83	0.68	−0.64	0.10	0.38	0.20
SE2	1	5	3.99	0.74	0.55	−0.84	0.10	1.52	0.20
SE3	1	5	4.00	0.81	0.66	−0.92	0.10	1.28	0.20
SE4	1	5	4.08	0.78	0.60	−1.01	0.10	1.42	0.20
All items			3.80	0.43	0.19	−0.11	0.10	0.51	0.20

**Table 4:** Response to self-regulated learning.

Item	Strongly Disagree		Disagree		Neutral		Agree		Strongly Agree	
	F	%	F	%	F	%	F	%	F	%
GS1	1	0.2	16	2.8	51	8.8	364	63.1	145	25.1
GS2	1	0.2	34	5.9	75	13.0	338	58.6	129	22.4
GS3	2	0.3	71	12.3	139	24.1	263	45.6	102	17.7
GS4	2	0.3	17	2.9	55	9.5	325	56.3	178	30.8
GS5	15	2.6	58	10.1	82	14.2	299	51.8	123	21.3
ES1	2	0.3	19	3.3	17	2.9	259	44.9	280	48.5
ES2	1	0.2	23	4.0	60	10.4	284	49.2	209	36.2
ES3	0	0.0	15	2.6	56	9.7	306	53.0	200	34.7
ES4	6	1.0	45	7.8	71	12.3	288	49.9	167	28.9
TS1	5	0.9	43	7.5	120	20.8	257	44.5	152	26.3
TS2	21	3.6	188	32.6	131	22.7	180	31.2	57	9.9
TS3	14	2.4	106	18.4	238	41.2	179	31.0	40	6.9
TS4	18	3.1	152	26.3	241	41.8	135	23.4	31	5.4
TM1	5	0.9	77	13.3	165	28.6	250	43.3	80	13.9
TM2	5	0.9	87	15.1	152	26.3	253	43.8	80	13.9
TM3	4	0.7	40	6.9	119	20.6	325	56.3	89	15.4
HS1	48	8.3	122	21.1	125	21.7	187	32.4	95	16.5
HS2	5	0.9	35	6.1	60	10.4	311	53.9	166	28.8
HS3	11	1.9	52	9.0	77	13.3	293	50.8	144	25.0
HS4	14	2.4	91	15.8	190	32.9	216	37.4	66	11.4
SE1	3	0.5	33	5.7	112	19.4	301	52.2	128	22.2
SE2	3	0.5	21	3.6	79	13.7	348	60.3	126	21.8
SE3	5	0.9	28	4.9	78	13.5	320	55.5	146	25.3
SE4	1	0.2	35	6.1	41	7.1	339	58.8	161	27.9

In order to evaluate students’ self-regulation, the scores obtained from the questionnaire were analyzed and shown in Table 4. All items adopted a 5-point Likert scale from 1 (strongly disagree) to 5 (strongly agree) with a midpoint of 3 (neutral). The collected data were then presented descriptively. The levels of students’ online SRL were displayed with their frequencies and proportions. For example, data obtained regarding Item GS3 “*I keep a high standard for my learning in my online courses*” shows that 263 (45.6%) respondents agreed and 102 (17.7%) strongly agreed. As illustrated in Table 4, in general, the rating distributions for each OSLQ item were concentrated in the “Neutral” and “Agree” options. This indicates that undergraduate students have a fairly high level of online SRL strategies. A possible reason for this result may be that students have been accustomed to engaging in setting high standards for their assignments, controlling their learning process, managing their behavior in pursuit of learning goals, implementing effective learning strategies, seeking help from lecturers, peers, and more knowledgeable people, focusing on the desired outcomes, and taking responsibility for their online courses in the time of COVID-19 [1,7,8].

After calculating the frequencies, the researcher checked whether there was a correlation between groups of variables. The researcher used bivariate correlation analysis to determine how strong the relationship is between groups of variables. Thus, correlation analysis was computed to investigate the association between measured variables. Data on Pearson's linear correlations between self-regulation constructs are displayed in Table 5.

**Table 5:** Correlation between SRL variables.

	GS	ES	TS	TM	HS	SE
Goal Setting	–	0.351⁎⁎	0.476⁎⁎	0.586⁎⁎	0.324⁎⁎	0.414⁎⁎
Environment Structuring		–	0.287⁎⁎	0.281⁎⁎	0.287⁎⁎	0.352⁎⁎
Task Strategies			–	0.597⁎⁎	0.443⁎⁎	0.449⁎⁎
Time Management				–	0.383⁎⁎	0.447⁎⁎
Help-Seeking					–	0.462⁎⁎
Self-Evaluation						–

∗∗ *p*<0.01 (2-tailed)

Table 5 summarizes six indicators. The level of confidence is presented below the table. Statistical analysis indicated low to high, positive correlations between the SRL behaviors. The correlation values ranged from 0.281 to 0.597 with a *p*-value of less than 0.01. The highest correlation appeared between Time Management and Task Strategies (*r*=0.597; *p*<0.01) and the lowest was found both between Environment Structuring and Task Strategies (*r*=0.287; *p*<0.01) and Environment Structuring and Time Management (*r*=0.287; *p*<0.01). In addition, the second-highest relationship was found between Goal Setting and Time Management (*r*=0.586; *p*<0.01) as this was higher than the 0.50 recommended by Cohen [9]. It can be stated that the way online learners manage their time is strongly associated with their use of task strategies and goal-setting behavior. This reflects that students who set goals and complete tasks in a remote learning environment tend to manage their time more appropriately. Additionally, students who structure their distance learning environment tend to have good time management skills and use different task strategies in online classes.

## 3 Experimental Design, Materials, and Methods

To fulfill the objective, the researcher employed the cross-sectional method. This dataset focused on the self-regulation skills of undergraduate students in online and blended learning environments throughout the COVID-19 crisis. Due to the lack of a sampling frame, the sampling method used to recruit the respondents was a mixture of purposive and snowballing. This sampling technique was employed because it allows the researcher to invite respondents who will provide the most valuable information [10]. By adopting this non-probability sampling technique, the researcher gained a deeper understanding of students’ perceptions of SRL during remote teaching and the factors influencing their perceptions. The inclusion criteria were: (i) Indonesian full-time undergraduate students, (ii) having a mobile device and access to the Internet, and (iii) enrolled in the blended courses. To collect data, an online questionnaire was distributed to the target sample via Google Forms. The setting in Google Forms was set to prevent students from taking the questionnaire more than once. A link to access the scale was sent to students and WhatsApp groups by lecturers and was available for a period of 4 weeks. Students were then encouraged to share the survey link with other peers at the same university, so that the survey could reach as many respondents as possible. All respondents came from UNJ, a public university founded in mid-1964. They could come from any faculty and were not limited to one study program.

Informed consent was obtained from all the respondents and the survey was approved by the ethics committee of Universitas Negeri Jakarta prior to data collection. All procedures performed in the study were in agreement with the ethical standards. Following approval from the IRB, the researcher scheduled a time to meet the lecturers. The researcher then conveyed the research objectives to the lecturers and invited their students to participate in the survey. They were asked to disseminate an online invitation on behalf of the researcher to all students they teach who are at least 18 years of age or older. The invitation included a link to the online survey. Invitations to complete the questionnaire were delivered in the last week of the course to ensure that students could reflect on their actual self-regulation strategies during distance learning. Before answering the questionnaire, all students were briefed on the goal of the study, data confidentiality, and their right to stop participating during the survey. Once clicking on the link, participants were automatically directed to the front of the questionnaire which presented the participant information letter. After giving consent, respondents completed the survey voluntarily and could withdraw at any time without any negative consequences. Students received no compensation for their participation in the study. They were aware that any data they provided would not affect their final grade. All questionnaires were filled out anonymously and kept confidential so that respondents could answer questions honestly.

Data were collected from May to June 2023, using the OSLQ designed by Barnard et al. [1] and available in the Mendeley repository [2]. Permission was obtained via email from the corresponding author to adapt the scale for the purposes of the current study. Prior to data collection, the original English version of the questionnaire was translated into Bahasa Indonesia. The OSLQ consisted of 24 close-ended questions on a five-point rating scale with a range of responses of 1 to 5 (“5” strongly agree, “1” strongly disagree) with 3 as a neutral midpoint. All items on the OSLQ are positively phrased. The questionnaire comprised two parts; the first part comprised 15 items focusing on socio-demographic variables. The second part included 24 items measuring students’ self-regulation skills. The items are presented in the OSLQ as statements, for instance, “*I set goals to help me manage study time for my online courses*”, “*I find a comfortable place to study*”, and “*If needed, I try to meet my classmates face-to-face*”. Before answering the 24 questions taken from OSLQ, respondents reported demographic information (e.g., faculty, gender, academic year, age, learning time before and during COVID-19, number of siblings, location of residence, source of tuition fees, cumulative GPA, daily internet usage, the purpose of internet usage, devices often use, parental education, and parental occupation). The respondents spent about 15–20 minutes completing the questionnaire. The maximum and minimum scores for the entire scale were 120 and 24, respectively. The higher scores earned by students demonstrate better self-regulation in hybrid learning, and vice versa. All items were made compulsory to be completed before submission; thus, no information was missing. As no further responses were received, the survey was then closed in the second week of June.

Due to the impact of COVID-19, most universities and educational institutions generally adopted an online teaching model. To explore the SRL situation, the researcher recruited undergraduate students registered in blended learning during the second term of the 2022/2023 academic calendar. The target respondents were from a state university located in Jakarta. To gather the data, the OSLQ was then administered online to active students. All participating students came from 8 different faculties, from a universe of 29,811 undergraduates at UNJ. To calculate an adequate sample size for a research study, the Taro Yamane [11] formula with a ninety-five percent confidence level was employed. According to calculations, the minimum number of respondents required was found to be 395. It should be noted that there were 849 clicks on the survey link at the end of the data collection process. Unfortunately, only 577 respondents filled out the questionnaire, the resulting response rate was 67.96%. This number has exceeded the recommended minimum number for the sample size [11].

All responses from respondents were processed manually. Data validation was carried out by checking duplicate email addresses, completeness, and quality of survey answers (whether the data met participation criteria, e.g. respondents were at least 18 years old). If duplicate email addresses and missing data were found (e.g., invalid answers or unanswered questions) and the respondent was less than 18 years old, the data were not included in the data analysis. It is noteworthy that the researcher did not apply an imputation procedure. Finally, 577 valid responses were received in spreadsheet formats. The researcher then exported them as an Excel file and imported them into the SPSS software for further analysis. In order to reveal the frequency of students’ use of different types of SRL strategies during hybrid learning, descriptive statistics were employed. The Pearson correlation analysis was utilized to assess any correlations between OSLQ constructs. This dataset was presented in raw and analyzed data forms and can be found in the Mendeley repository [2].

## Limitations

Data were collected from a single university only, thereby limiting the generalizability of the results obtained and may not represent all university students in Indonesia. It is worth noting that all participants in the current study lived in Jakarta; thus, it is possible that the results are influenced by the Indonesian sociocultural context. Therefore, further investigation in other countries or cultural contexts may shed more light on the interactions between online self-regulation variables. Additionally, to increase the validation of research findings, future research may need to use random sampling to ensure adequate representativeness of the sample. Furthermore, the results of the present study may be exclusive to online learners, as learners in other learning environments may demonstrate different self-regulation skills. Thus, future research should replicate this study and compare it with students in other learning environments. Another direction for further research is to combine quantitative and qualitative research designs. Qualitative studies through in-depth interviews, think-aloud strategies, and observations can be adopted and combined with questionnaires to help educators gain a more holistic understanding of students’ self-regulation during distance education. Given the current findings, the use of experimental designs on a large scale should also be investigated to improve students’ self-regulation skills to a satisfactory level.

## Ethics Statement

This work was conducted in accordance with the Declaration of Helsinki and was approved by the Research Ethics Committee of Universitas Negeri Jakarta, Indonesia (826/UN39.14/PT/XI/2022). Informed consent [2] was displayed on the front page of the questionnaire. After the student clicked the “Next” button in the Google Form, he/she was deemed to have read the information about the survey and agreed to voluntarily participate in the survey. To guarantee anonymity and confidentiality, no personal data of the respondents were gathered.

## CRediT authorship contribution statement

**Irwanto Irwanto:** Conceptualization, Methodology, Validation, Software, Data curation, Writing – original draft, Visualization, Investigation, Supervision, Writing – review & editing.

## Data Availability

Data on undergraduate students’ self-regulation in online and blended learning environments during the COVID-19 pandemic in Indonesia (Original data) (Mendeley Data).
